# Supplementary material for: Modelling Skylarks (Alauda arvensis) to Predict Impacts of Changes in Land Management and Policy: Development and Testing of an Agent-Based Model
Source: PLoS One. 2013 Jun 6;8(6):e65803. doi: 10.1371/journal.pone.0065803 (PMC3675089; doi:10.1371/journal.pone.0065803)
Supplement: Supporting Information S4 — The skylark ODdox as a zipped archive. (ZIP) [file pone.0065803.s004.zip › Skylark_ODdox/maperrormsg_8cpp.html]

ALMaSS Skylark ODdox: maperrormsg.cpp File Reference


ALMaSS Skylark ODdox
 2.0


- Main Page
- Related Pages
- Classes
- Files

- File List
- File Members

Macros |
Variables

maperrormsg.cpp File Reference

`#include <cstdio>`  
`#include <time.h>`  
`#include <string>`  
`#include <cstdlib>`  
`#include "maperrormsg.h"`

|  |  |
| --- | --- |
| Macros | |
| #define | \_CRT\_SECURE\_NO\_DEPRECATE |

|  |  |
| --- | --- |
| Variables | |
| class MapErrorMsg \* | g\_msg |

---

## Macro Definition Documentation

#define \_CRT\_SECURE\_NO\_DEPRECATE

---

## Variable Documentation

class MapErrorMsg\* g\_msg

Referenced by Landscape::AddBeetleBanks(), LE\_TypeClass::BackTranslateEleTypes(), LE\_TypeClass::BackTranslateVegTypes(), Landscape::BorderAdd(), Landscape::BorderNeed(), Skylark\_Population\_Manager::BreedingPairsOutput(), Skylark\_Population\_Manager::BreedingSuccessProbeOutput(), Landscape::ChangeMapMapping(), Farm::CheckRotationManagementLoop(), FarmManager::ConnectFarm(), FarmManager::CreateFarms(), CropRotation::CropRotation(), Calendar::DayLength(), RodenticideManager::DoPlaceBait(), Landscape::DumpMap(), Landscape::DumpMapGraphics(), SkTerritories::DumpMapGraphics(), Landscape::DumpMapInfoByArea(), Configurator::DumpSymbols(), Landscape::DumpTreatCounters(), Landscape::EventDump(), Landscape::EventtypeToString(), Configurator::ExtractString(), probe\_data::FileAppendOutput(), CropData::FindCropNum(), Skylark\_Population\_Manager::FledgelingProbeOutput(), Landscape::ForceArea(), RasterMap::Get(), CropRotation::GetFirstCrop(), CropRotation::GetNextCrop(), Landscape::IncTreatCounter(), MovementMap::Init(), Farm::InitiateManagement(), UserDefinedFarm::InvIntPartition(), Landscape::Landscape(), Farm::LeSwitch(), Farm::Management(), Landscape::NewElement(), probe\_data::OpenFile(), probe\_data::OpenForAppendToFile(), Skylark\_Population\_Manager::OpenTheBreedingPairsProbe(), Skylark\_Population\_Manager::OpenTheBreedingSuccessProbe(), Population\_Manager::OpenTheCIPEGridOutputProbe(), Population\_Manager::OpenTheMonthlyRipleysOutputProbe(), Population\_Manager::OpenTheReallyBigProbe(), Population\_Manager::OpenTheRipleysOutputProbe(), Landscape::OrchardBorderAdd(), Configurator::ParseCfgLine(), Pesticide::Pesticide(), Landscape::PolysDump(), Landscape::PolysValidate(), Landscape::PolytypeToString(), SkTerritories::PreFillQualGrid(), RasterMap::RasterMap(), VegElement::ReadBugPercentageFile(), Landscape::ReadPolys(), Farm::ReadRotation(), Configurator::ReadSymbols(), Farm::RemoveField(), Pesticide::SavePPM(), Configurator::SetCfgBool(), Configurator::SetCfgFloat(), Configurator::SetCfgGatekeeper(), Configurator::SetCfgInt(), Configurator::SetCfgStr(), CropData::SetVegNum(), Configurator::ShowIdType(), Skylark\_Female::st\_Finding\_Territory(), Skylark\_Female::st\_Laying(), Skylark\_Female::st\_MakingNest(), Farm::TranslateCropCodes(), LE\_TypeClass::TranslateEleTypes(), LE\_TypeClass::TranslateVegTypes(), Landscape::UnsprayedMarginAdd(), UserDefinedFarm::UserDefinedFarm(), Landscape::VegDump(), CropData::VegTypeToCurveNum(), Landscape::VegtypeToString(), Landscape::Warn(), Weather::Weather(), and Landscape::~Landscape().


- CJT
- MSVC
- ALMaSS Working Source
- Landscape
- maperrormsg.cpp
- Generated on Thu Jan 10 2013 13:15:35 for ALMaSS Skylark ODdox by
   1.8.1.1
